# Mental Health Care at the Borders of Youth, Trauma and Migration: An Exploratory Survey of Mental Health Practitioners

**DOI:** 10.1111/inm.70365

**Published:** 2026-09-25

**Authors:** Richard West, Wanyu Flora Yennyuy, Carmel Bond

**Affiliations:** ^1^ Sheffield Hallam University Sheffield UK; ^2^ Humber Teaching NHS Foundation Trust Mental Health Nurse Hull UK

**Keywords:** adolescents, applied health research, asylum seekers, exploratory survey, mental health, mental health practitioners, qualitative, refugees

## Abstract

Young asylum seekers and refugees often present with complex mental health needs arising from forced migration, trauma and ongoing legal uncertainty. Existing evidence indicates that mental health practitioners frequently report lower confidence in assessment and intervention when working with asylum seekers and refugees; however, most research to date has focused on adult populations. An exploratory qualitative survey containing open‐ended questions was distributed to mental health practitioners between 1 June and 1 September 2025. Interpretive description guided the inductive analysis of free‐text responses from 116 UK practitioners, of whom over half reported low confidence in working with young asylum seekers and refugees. Three key themes were identified: development of professional confidence, cultural responsiveness and constrained agency. Despite a strong commitment to equitable care, low confidence hinders effective practice. Targeted training for mental health practitioners and protected supervision could enhance practitioner confidence and care quality.

## 1 Introduction

Global migration is increasing, young asylum seekers and refugees face complex mental health needs from forced migration, trauma and legal uncertainty (Kien et al. 2019). Internationally, over 50 million children comprise ~40% of displaced populations, underscoring urgent multi‐service demand (Save the Children 2024; UNICEF 2024). In the UK, asylum claims from those under 25 reached nearly 28 000 in 2025 (Home Office 2025). These young people exhibit elevated post‐traumatic stress disorder, anxiety and depression rates amid developmental disruption. International evidence points to intensifying pressure on young people's' mental health services where there is a growing gap between demands and availability of care (Pollard and Howard 2021; Blackmore et al. 2020; World Health Organization 2025). In this paper the term young asylum seeker and refugee is defined as young person under 25 years old. An asylum seeker is an individual who has applied for international protection and is awaiting a decision on their claim, whereas a refugee is someone whose claim has been recognised legally. Although both groups may have experienced forced displacement and trauma, asylum seekers often face greater uncertainty due to the ongoing asylum process.

As a large part of the multi‐disciplinary team, mental health practitioners are central to holistic wellbeing and mental health recovery yet report under‐preparation in addressing the cultural‐linguistic barriers and mistrust of services associated with this vulnerable population (Peñuela‐O'Brien et al. 2022). Supporting these young people often evokes professional frustration and feelings of powerlessness as nurses navigate their roles amid fragmented services and resource constraints (Trueba et al. 2023; Gabrielsson et al. 2022). If left unaddressed, low practitioner confidence, coupled with access barriers such as convoluted referrals and stigma risks suboptimal therapeutic alliances, and ultimately poorer outcomes, potentially exacerbating future service strains (West et al. 2025). Despite the increased mental health needs of young asylum seekers and refugees and given the central role that mental health practitioners play in recognising, assessing and responding to the needs of young asylum seekers and refugees, understanding their experiences and perceived preparedness is essential. Existing research has largely focused on the experiences and mental health needs of young asylum seekers themselves (West et al. 2025; Pollard and Howard 2021), with comparatively little attention paid to the perspectives of the practitioners responsible for delivering care. Exploring practitioners' experiences may help identify barriers and enablers to culturally responsive, developmentally appropriate mental healthcare and inform future education, supervision and service development.

## 2 Background

Children and young people comprise a substantial proportion of forcibly displaced populations worldwide, exhibiting elevated rates of mental illness that signal heightened future demand for mental health services (West et al. 2025; Blackmore et al. 2020). Mental health practitioners are pivotal in supporting young people but report inadequate preparation in caring for this demographic (Peñuela‐O'Brien et al. 2022). A lack of targeted training often leaves practitioners underconfident, compounded by access barriers like communication challenges and concern over uncertain futures for this group (Pollard and Howard 2021).

Practitioners' understanding of the issues surrounding young asylum seekers and refugees may have implications beyond immediate care. Evidence shows that underprepared mental health nursing leads to fragmented support for these young people, which exacerbates lifetime risks of social exclusion, diminished quality of life, and curtailed educational and employment prospects (West et al. 2025). These young people also endure protracted trauma during critical developmental windows, heightening vulnerabilities to post‐traumatic stress disorder, anxiety, depression and disrupted psychosocial growth (Blackmore et al. 2020; Kien et al. 2019). In the UK, asylum claims from those under 25 have manifested this global crisis locally—straining services ill‐equipped for multifaceted needs like traumatic migration, unaccompanied status and cultural barriers (UNICEF 2024; Khanom et al. 2021; Kien et al. 2019).

Trueba et al. (2023) discuss disparities between asylum seekers' needs and fragmented mental health services, including convoluted referrals, communication impasses and systemic stigma. Peñuela‐O'Brien et al.'s (2022) European review echoes practitioner strains from legal and/or emotional impacts (as well as cultural‐linguistic gaps); systems analyses reveal delayed engagement, mismatched pathways and time constraints undermining trust in services (Robertshaw et al. 2017).

Gabrielsson et al. (2022) offer insights into young asylum seekers and refugees' experiences, highlighting the role of mental health practitioners in affirming immediate feelings of safety and trust‐building. On the other hand, these authors also acknowledge enduring limitations of the asylum system and asylum reform, as well as access to long‐term therapy for those seeking asylum who are younger in age. West et al. (2025) further confirm high youth mental health burdens. However, while these works offer some insights into mental health practitioners' role limitations with young asylum seekers and refugees, primary research from the perspective of mental health practitioners in caring for this population is absent from the literature.

Currently, mental healthcare for young asylum seekers remains inconsistent across UK health services. Community and inpatient units incorporate ad‐hoc cultural check‐ins or referrals, but these rely on individual initiative rather than standardised, trauma‐informed protocols (Trueba et al. 2023; Peñuela‐O'Brien et al. 2022). Moreover, no nationwide mandate exists for routine confidence assessment or culturally responsive training. Assessing practitioner confidence may help identify gaps in knowledge, training, or supervision which would help organisations develop safe and equitable mental healthcare (Home Office 2025). Recent work has conceptualised nurse‐led responses to these challenges through capacity‐building approaches; for example, Teixeira‐Santos and de Abreu (2025) describe the development of a structured mental health nurse–led training programme to equip non‐health professionals working with young asylum seekers to recognise distress, refer appropriately and sustain their own well‐being. By strengthening practitioners' knowledge and skills, workforce capacity‐building initiatives may improve practitioners' ability to establish trusting therapeutic relationships working through feelings of mistrust that can influence engagement with mental health services (Pollard and Howard 2021). Therefore, understanding factors that might enable practitioner confidence and the development of cultural sensitivity is essential for embedding feasible, equitable practices into routine care.

Given the scant literature on mental health practitioners' experiences of working with young asylum seekers and refugees, there is a need to gain knowledge of what is needed to improve practitioner preparedness, and to support positive outcomes for this population in the future. The current study goes some way to addressing this gap.

## 3 Methods

### 3.1 Aim

To explore mental health practitioners' experiences of working with young asylum seekers and refugees.

### 3.2 Study Design

The study employed an exploratory qualitative survey design. Interpretive description (Thorne 2016) informed the qualitative analysis of the free‐text responses. This was selected for its suitability in generating applied knowledge from practitioner free‐text responses within mental health contexts.

### 3.3 Setting, Participants and Recruitment

The study was conducted within mental health services, across multiple sites in the north of England. Respondents were eligible if they were a current practising mental health practitioner and had any previous or current experience of working with young asylum seekers and refugees. Participants were recruited via convenience and snowball sampling through professional networks and organisational newsletters (e.g., staff bulletins). Convenience and snowball sampling methods were appropriate for this preliminary exploratory qualitative study due to time constraints, lack of a defined population frame for UK mental health practitioners working with young asylum seekers, and the need for rapid data collection from accessible professionals via existing networks (Bryman 2012). The survey was distributed between 1 June and 1 September 2025.

### 3.4 Data Collection

Data was generated using a structured online survey, developed from, and building on, the findings of a scoping review (West et al. 2025). This approach informed item selection to ensure relevance and comprehensiveness. The survey included demographic questions such as age, gender, professional role and years of experience. It also included Likert‐scale items assessing practitioners' perceived confidence in recognising, assessing and supporting the mental health needs of young asylum seekers and refugees, particularly those presenting with severe mental health conditions, alongside open‐ended free‐text questions inviting respondents to elaborate on the factors influencing their confidence (see File S1 for survey questions). The latter formed the central focus for the analysis in this paper.

Participants were recruited because they had current or previous experience of working with young asylum seekers and refugees, ensuring that the free‐text responses reflected relevant practice‐based perspectives. Although the survey also generated descriptive quantitative data, the qualitative analysis focused on practitioners with experiential knowledge of the phenomenon under investigation. The professionally diverse sample enabled the exploration of shared patterns and variations in practitioners' experiences across mental health disciplines. The survey yielded 116 responses; however, as the total population of eligible practitioners was unknown, a response rate could not be calculated.

### 3.5 Data Analysis

Qualitative free‐text responses were analysed using interpretive description, as outlined by Thorne (2016). The aim was developed for this study, informed by a review of young asylum seekers and refugees (West et al. 2025). Analysis sought to generate practice‐relevant interpretive insights by examining patterns across participants' responses. This approach was selected to enable the generation of practice‐relevant recommendations. Following repeated immersion and familiarisation with the data, the first author generated initial codes and memos to explore patterns and relationships across participants' accounts. Emerging interpretive patterns were developed in collaboration through ongoing reflection and discussion with the second author, ensuring that the analysis remained grounded in the data while generating practice‐relevant insights consistent with interpretive description.

The process of theme identification was iterative and involved continuous reflection and dialogue between the researchers to ensure rigour and coherence in the analysis. Researchers (R.W., C.B. and F.W.) are experienced mental health practitioners, bringing a combined 45 years of professional experience across both adult and mental health clinical practice (including Child and Adolescent Mental Health) and academia. As such, the authors acknowledge their position as insiders in relation to the topic under investigation (Chavez 2008). This disciplinary background and insider positionality informed a nuanced understanding of care complexity and professional practice, while necessitating reflexive awareness of how prior knowledge and assumptions might shape interpretation (Patton 2001). One of the authors, F.W., has lived experience of being an asylum seeker and a qualified mental health nurse, so brought a wealth of valuable insight to the study.

The descriptive quantitative findings were used to contextualise the study sample and practitioners' reported confidence levels. The qualitative analysis and resulting interpretive patterns were derived exclusively from the free‐text survey responses.

### 3.6 Rigour and Transparency

Trustworthiness was maintained following Lincoln and Guba's (1985) criteria. Credibility was enhanced through sustained engagement with the data and repeated cycles of analysis and reflection. Dependability was supported by clear documentation of analytic decisions and procedures, ensuring an auditable trail. Confirmability was achieved through a reflexive approach that acknowledged the researchers' professional backgrounds as mental health practitioners and the potential influence of prior beliefs and assumptions on interpretation. The resulting themes therefore reflect not only the participants' accounts but also the interpretive relationship between the data and the researchers' positionality as practitioners and academics. Although the data were drawn from mental health practitioners in the United Kingdom, the findings may have relevance and potential transferability to other healthcare contexts.

### 3.7 Ethical Considerations

Ethical approval for this study was granted by Sheffield Hallam University Research Ethics Committee (ID: ER77953805). Participation was voluntary, and all participants provided informed consent before completing the survey. Participants could withdraw at any time, and no identifiable information was collected to maintain confidentiality. An optional incentive was provided to enhance engagement.

## 4 Results

The Consolidated Criteria for Reporting Qualitative Studies (COREQ) checklist was used for reporting; please see File S2 (Tong et al. 2007). In total, 116 participants completed the survey including 23 mental health nurses, 21 social workers, 24 psychiatrists, 14 mental health practitioners, 22 psychologists and 4 “other”. Participants were majority 90% female and 9% male. The participants reflected an ethnically diverse background. Detailed demographic data of participants are shown in Table 1. All participants reported current or previous experience of working with young asylum seekers and refugees. The majority considered supporting the mental health needs of this population to be an important aspect of their role and indicated that further guidance and training would be beneficial.

**TABLE 1:** Participant characteristics and demographics.

Characteristics	Participant responses (*n* = 116)
Gender	
Female	104
Male	10
Prefer not to say	2
Ethnicity	
White	78
Black/African/Caribbean	11
Asian	13
Mixed two or more ethnic groups	10
Other	4
Age	
Under 24	1
25–34	33
35–44	38
45–54	28
55 and over	15
Prefer not to say	1
Professional occupation	
Mental Health Nurse	23
Social Worker	21
Psychiatrist	24
Mental Health Practitioner (Self‐Identified)	14
Psychologist	22
Therapist	3
Other	9
Years qualified	
< 2	2
2–5	56
6–10	39
> 10	10
Prefer not to say	9

Participants were asked whether they had received any training to support the mental health needs of young asylum seekers and refugees. 38% (*n* = 44) reported having received guidance or training related to supporting the mental health needs of young asylum seekers and refugees, while 62% (*n* = 72) reported receiving no guidance or training.

Participants were asked to rate their perceived confidence in supporting the mental health needs of young adult asylum seekers/refugees with severe mental health conditions using a 5‐point Likert scale. Overall, 84 respondents rated their confidence as ‘low’ or ‘very low’.

The qualitative dataset comprised brief to moderately detailed free‐text responses generated across five open‐ended survey questions. Although the responses did not permit follow‐up probing. Analysis was undertaken across five open‐ended survey questions, with between 40 and 60 responses contributing to each question.

Three themes supported by 1 sub‐theme were identified as follows: (1) development of professional confidence, (2) cultural responsiveness and (3) constrained agency (Table 2).

**TABLE 2:** Description of main themes and subthemes.

Theme	Description
Developing professional confidence	Confidence linked to level of professional experience and previous exposure to young asylum seekers Lower confidence linked to uncertainty over role clarity and pathways of care Navigating complex systems and difficulties in identifying specialist pathways
Subtheme: strategies	Need for targeted training Protected supervision time
Cultural responsiveness	Increased empathy due to own lived experiences which enables cultural capacity and sensitivity Reflections on continued professional development
Constrained agency	Language & engagement issues—barriers to responsiveness and healthcare professionals' agency Feelings of frustration over system constraints (healthcare and immigration/asylum)—linked to sphere of control and lack of influence over perceived positive changes

### 4.1 Theme 1: Developing Professional Confidence

This theme highlights the variable confidence of practitioners expressed when working with young asylum seekers and refugees, and the underlying factors influencing both high and low confidence levels.

Some respondents linked their levels of prior professional experience and exposure to diverse client groups

I have worked in CAMHS for over 14 years, and I have worked with many different cultures with many different mental health disorders. (Mental Health Nurse 1)

Other practitioners described uncertainty about how best to assess and respond to the mental health needs of young asylum seekers and refugees.

I would be unsure how to approach and identify their mental health needs. (Social Worker)

Confidence was also affected by uncertainty around role boundaries. Some practitioners perceived elements of asylum support as extending beyond their professional remit:

At times, I am unsure where my role begins and ends. (Mental Health Nurse 2)

The complexity of some young people's circumstances appeared to blur professional roles and responsibilities, leaving practitioners uncertain about their place within broader systems of care. Several respondents questioned whether supporting asylum processes or housing issues fell within their role.

Finally, participants described how overlapping vulnerabilities, such as trauma histories, language barriers, and systemic obstacles, further complicated care and influenced their confidence:

Supporting young asylum seekers' mental health is incredibly tough. Language barriers and deep‐seated trauma from their past are huge hurdles. On top of that, navigating the complex asylum system and finding specialised services in the UK often makes it harder for them to get the help they need. (Mental Health Nurse 3)

Collectively, these accounts illustrate professional confidence as a dynamic and context‐dependent construct rather than a fixed attribute. Practitioners' confidence appeared to fluctuate in response to their prior experience, perceived competence, systemic barriers and clarity of role. Importantly, some participants described a reflective awareness of their limitations, which, rather than representing simple deficit, hinted at a developing capacity for professional growth. This self‐awareness may serve as a foundation for cultivating greater confidence through experience, supervision and collaborative practice.

#### 4.1.1 Sub Theme: Strategies

Participants described a desire for organisational support and strategies that could help them develop and sustain professional confidence when working with young asylum seekers and refugees. Access to protected time for supervision, reflection, and peer discussion was viewed as particularly valuable for consolidating learning and emotional resilience:

Regular seminars and reflective spaces would support ongoing learning. (Psychiatrist)

Practitioners consistently said that their confidence would be strengthened by targeted training and structured organisational support. They expressed a need for specific learning opportunities focused on both the asylum process and the cultural contexts of the young people they encounter:

Training on asylum processes, legal frameworks, and their impact. (Mental Health Nurse 1)

Training on cultural knowledge relating to specific migrant groups. (Psychologist)

Across these accounts, participants framed confidence as something that can be developed collaboratively rather than acquired individually. Their reflections point to a shared belief that access to appropriate training, reflective spaces, and sustained organisational support can help overcome some of the barriers previously described in their work with young asylum seekers and refugees. This suggests that confidence evolves through experience, discussion and the availability of supportive structures within practice settings.

### 4.2 Theme 2. Cultural Responsiveness

Culturally responsive care entails remaining alert to, and acting congruently with, individuals' unique beliefs and conventions rooted in their cultural backgrounds—at both personal and policy levels. Lived migration experience enabled some practitioners to empathise more confidently with young asylum seekers and refugees:

I am myself a migrant, so I feel I understand feeling of displacement, home sickness and fear about my future considering increase in riots and negativity towards migration in the UK. (Mental Health Nurse 5)

This nurse's background fostered empathy amid 2025 anti‐migration protests, heightening young people's settlement uncertainties.

Personal exposure beyond work further built cultural confidence. Social activities with diverse groups enhanced practitioners' abilities:

I have built confidence through my social life. (Mental Health Nurse 6)

Conversely, training contexts could constrain responsiveness:

I feel that when we talk about ‘trauma’ we look at this through a very ‘safe’ British perspective. (Social Worker)

Here, the participant critiques Western diagnostic frameworks that pathologise trauma responses rather than viewing them as understandable reactions to abnormal circumstances.

Practitioners proposed practical solutions from experience:

Using trauma‐informed approaches – e.g., using knowledge and themes around culture, identity, religion, and language that the young person already knows and is familiar with, rather than imposing unfamiliar concepts. (Psychiatrist)

Overall, cultural responsiveness emerges through lived experience, personal and professional, coupled with critical awareness of UK mental health practice limitations.

### 4.3 Theme 3. Constrained Agency

Constrained agency emerged as practitioners' pervasive sense of limited power to meaningfully improve young asylum seekers' and refugees' mental health, often due to external priorities like immigration status overriding therapeutic gains. One social worker captured this:

Ongoing anxiety linked to immigration status cannot be resolved by therapy alone. (Social Worker)

Some of the challenges to the mental health of young asylum seekers were beyond the control of the practitioners, for example ongoing issues asylum status. Unlike young refugees, whose legal status has been recognised, young asylum seekers often remain in long periods of uncertainty while awaiting decisions on their asylum claims. This at times led to a sense of frustration of what they can realistically achieve when working with young asylum seekers, which in turn could diminish the sense of confidence in the value of their interactions. This led to a sense of helplessness in the responses:

Asylum seekers and military professionals don't get the help and treatment they need. (Mental Health Nurse 7)

Organisational and systemic issues thus impeded access to essential care. Yet variation existed; some resisted deferring interventions amid instability:

Many therapists or professionals will take the view of ‘if there is no stable home then we will not do any intervention’ however these young people can be drifting without stable bases for many years, and they still deserve intervention and still massively benefit from it. (Mental Health Practitioner)

This quote highlights intra‐professional tensions, affirming mental health support's value despite precarity. While echoing the initial theme in terms of role boundary uncertainties, constrained agency uniquely manifests as systemic powerlessness—practitioners' moral distress arises not from personal skill deficits, but from organisational omissions and structural harms that undermine youth care amid broader adversities (e.g., prolonged instability). Recognising this interplay underscores the need for policy‐level interventions alongside confidence‐building strategies, ensuring mental health practitioners can act equitably within constrained contexts.

## 5 Discussion

This study aimed to explore mental health practitioners' experiences of working with young asylum seekers and refugees. Study findings show that, overall mental health practitioners report low self‐confidence when working with young asylum seekers and refugees. Findings also indicate that low practitioner confidence is not simply an individual characteristic but is produced through interactions between professional preparedness, cultural responsiveness, organisational constraints, role ambiguity, and the structural realities of working with young asylum seekers and refugees. This reported low confidence is in line with recent work that found 2 in 5 psychiatrists believed they had sufficient knowledge to work competently with adult asylum seekers and refugees (Tham et al. 2025). Unlike Tham's knowledge deficits fostering negative attitudes, our findings nuance low confidence as dynamic: prior exposure served to build resilience, yet complex trauma and language barriers eroded it, risking disengagement cycles documented by West et al. (2025). Perhaps a more useful framing would be professional preparedness as there was variability in this which might reflect the benefit of length of experience in mental health over a prolonged period accruing beneficial strategies. From the responses confidence is a factor that can be modified through training delivered by lived experienced practitioners, working closely with interpreters and ultimately retaining staff, allowing practitioners build the experience needed to work effectively with young asylum seekers and refugees.

The current study's findings relating to the development of professional preparedness align with that of Yada et al. (2020), who linked uncertainty in psychiatric nursing and loss of role clarity as key factors associated with reduced self‐efficacy among psychiatric nurses. Practitioners in our study echoed this: “unsure where my role begins and ends”, as asylum seeking and housing status blurred professional role boundaries. This resonates with Procter's (2005) earlier work on emergency mental health nursing with refugees and asylum seekers, which emphasised the importance of role clarity and trust in sustaining therapeutic engagement during periods of acute distress and cautioned that ambiguity in professional positioning can undermine both nurse confidence and care continuity. Both Yada and Procter discuss how uncertainty can limit cultural responsiveness. Uncertainty arises when practitioners lack familiarity with a young person's cultural context, which can undermine confidence and limit cultural responsiveness. These findings suggest that confidence in this context is shaped not only by role clarity but also by practitioners' perceived cultural competence when engaging with young asylum seekers.

Cultural responsiveness requires curiosity and risks “getting it wrong”. Lived migration fostered empathy, whereas on the other hand, existing cultural context, e.g., “safe British trauma lens” (quote from survey) limited responsiveness. Addressing this uncertainty through targeted training, reflective supervision, and clearer pathways may therefore be a key mechanism for strengthening cultural responsiveness, rather than treating it as another proficiency or task. Findings relating to cultural responsiveness echoes Peñuela‐O'Brien et al.'s (2022) European review of cultural‐linguistic strains (adult‐focused), while also uniquely highlighting youth‐specific needs, for instance, practitioners called for contextual depth and greater understanding of the asylum processes.

Constrained agency manifested as profound systemic powerlessness, exemplified by the social worker's assertion that ongoing anxiety linked to immigration status cannot be resolved by therapy alone. This echoes Gabrielsson et al. (2022) delineation of nurses' role limits in youth care, capable of fostering immediate safety and trust, yet powerless to enact systemic change. In contrast to West et al.'s (2025) emphasis on client reluctance to engage services, practitioners' perceived ineffectiveness here actively perpetuates disengagement cycles. Notably, variation emerged, with some resisting gatekeeping by affirming that young people deserve and benefit from intervention despite instability.

Collectively, the current study findings reveal professional confidence as a multi‐level phenomenon: personal factors related to prior experience and role clarity, cultural gaps in contextual understanding and responsiveness, and structural limits on agency. This study's exclusive youth focus fills critical gaps in the predominantly adult‐biased literature, where mental health practitioners' perspectives remain notably scarce. While low practitioner confidence risks reinforcing documented mistrust and access barriers (Pollard and Howard 2021), the current study provides insight into potential modifiability through targeted training and supervision, which may have clear practice implications, such as embedding trauma‐migration modules and protected reflective spaces. However, further work is needed to examine how national guidelines are being implemented in practice, particularly in settings where resource constraints may undermine equitable access. Furthermore, future longitudinal or realist review approaches would be useful in exploring whether targeted training, embedded specialist roles, or structured supervision models effectively translate (and how) into improvements in practitioner confidence and result in improved care quality.

## 6 Limitations

Self‐selection bias may have influenced participation, with individuals valuing reflective practice or holding favourable views of young asylum seekers and refugees more likely to respond, potentially narrowing perspective diversity. However, the sample demographics, spanning nurses, allied professionals and psychiatrists, offer a representative cross‐section of the UK mental health workforce.

Data derived exclusively from Northern England practitioners reflects regional asylum service contexts rather than national or international norms. This study nonetheless makes a substantive contribution as the first exploration of UK mental health practitioners' confidence with young asylum seekers and refugees.

As an exploratory qualitative survey, this study relied on free‐text responses rather than interviews. Although this enabled the inclusion of a relatively large and diverse sample of mental health practitioners, individual responses were generally brief and did not allow for probing or clarification. Consequently, the findings should be interpreted as practice‐oriented insights into practitioners' experiences rather than an in‐depth exploration of individual perspectives.

## 7 Conclusions

This study represents the first exploration, to our knowledge, of UK mental health practitioners' confidence in supporting young asylum seekers and refugees, revealing pervasive low confidence arising from unfamiliar trauma presentations, role boundary uncertainties, systemic navigation challenges, language barriers, and legal and cultural and organisation constraints. Despite these hurdles, practitioners affirmed significant positive impact on young people, even during periods of instability. Targeted education should combine knowledge of asylum processes with developmentally appropriate, trauma‐informed care for children and young people, cultural responsiveness, and protected supervision. Such approaches may strengthen practitioners' confidence while improving the quality and responsiveness of mental healthcare for young asylum seekers and refugees.

## 8 Relevance for Clinical Practice

Mental health practitioners should integrate asylum seeker‐specific modules into education and training, employing trauma‐informed case studies to address unfamiliar presentations. Mental health practitioners should also receive structured education on asylum processes, developmentally appropriate trauma‐informed care, interpreter‐mediated communication, referral pathways and professional role boundaries. Protected supervision may also foster role clarity amid boundary uncertainties, while reflective spaces cultivate cultural responsiveness through empathy and contextual curiosity. To counter constrained agency, services must develop migration process resources and advocate systemic navigation tools, enabling young people to better navigate unfamiliar systems. Ultimately, improving care requires more than increasing individual practitioner confidence; it depends on commitment to education, reflective supervision and culturally responsive systems that enable practitioners to provide equitable mental healthcare for young asylum seekers and refugees.

## Author Contributions

Richard West, Carmel Bond, Flora Yennyuy: Conceptualization, Ideas; formulation or evolution of overarching research goals and aims; Methodology, Development or design of methodology; creation of models, Formal analysis, Application of statistical, mathematical, computational, or other formal techniques to analyse or synthesise study data, Resources, Provision of study materials, reagents, materials, patients, laboratory samples, animals, instrumentation, computing resources, or other analysis tools, Data Curation, Management activities to annotate (produce metadata), scrub data and maintain research data (including software code, where it is necessary for interpreting the data itself) for initial use and later reuse, Writing – review and editing, preparation, creation and/or presentation of the published work by those from the original research group, specifically critical review, commentary or revision – including pre‐or post‐publication stages, Visualisation, Preparation, creation and/or presentation of the published work, specifically visualisation/data presentation. Richard West, Carmel Bond: Software, Programming, software development; designing computer programmes; implementation of the computer code and supporting algorithms; testing of existing code components, Validation, Verification, whether as a part of the activity or separate, of the overall replication/reproducibility of results/experiments and other research outputs, Investigation, Conducting a research and investigation process, specifically performing the experiments, or data/evidence collection, Investigation, Conducting a research and investigation process, specifically performing the experiments, or data/evidence collection, Writing – Original Draft, Preparation, creation and/or presentation of the published work, specifically writing the initial draft (including substantive translation), Project administration, Management and coordination responsibility for the research activity planning and execution. Richard West: Funding acquisition, Acquisition of the financial support for the project leading to this publication. Carmel Bond: Supervision, Oversight and leadership responsibility for the research activity planning and execution, including mentorship external to the core team.

## Funding

This work was supported by the National Institute for Health and Care Research.

## Ethics Statement

Ethical approval was granted by Sheffield Hallam University Research Ethics Committee (ID: ER77953805).

## Conflicts of Interest

The authors declare no conflicts of interest.

## Supporting information


**File S1:** inm70365‐sup‐0001‐SupinfoS1‐S2.docx.
**File S2:** COREQ 32‐item Checklist.

## Data Availability

Data are available on reasonable request due to privacy and ethical restrictions.
