# Supplementary material for: Mental Health Care at the Borders of Youth, Trauma and Migration: An Exploratory Survey of Mental Health Practitioners
Source: Int J Ment Health Nurs. 2026 Sep 25;35(5):e70365. doi: 10.1111/inm.70365 (PMC13615293; doi:10.1111/inm.70365)
Supplement: Supplementary file 1 [file INM-35-0-s001.docx]

### **Supplementary File 1**

**Questions of Survey**

Q1 | Consent

Q2 | What best describes your gender?

Q3 | What best describes your ethnic origin?

Q4 | What is your age group?

Q5 | What is your current occupation?

Q6 | How many years have you been a qualified healthcare practitioner?

Q7 | In your current role, have you worked with young refugee or asylum seekers? Yes/No

Q8 | Importance of young asylum seeker mental health as an issue, Likert 5-point scale

Q9 | How would you rate your confidence supporting mental health needs of young asylum seekers , Likert 5-point scale

Q10 | What factors have influenced your confidence? Free text

Q11 | Challenges faced when supporting young asylum seekers Free text

Q12 | Have you received guidance or training? Yes/No

Q13 | Strategies or approaches you found helpful? Free text

Q14 | Training, resources, or support needed Free text

Q15 | Suggested changes to improve mental health support for young asylum seekers Free text

Q16 | Do you think further guidance is needed? Yes/No

Q17 | Email for voucher draw (Optional)

### **Supplementary File 2 - COREQ 32-item Checklist**

**Domain 1: Research team and reflexivity**

**Personal characteristics**

| **No.** | **Item** | **Where reported in your manuscript** |
| --- | --- | --- |
| 1 | Interviewer/facilitator | **N/A** – no interviews/focus groups (online survey free-text) *(Method 3.4)* |
| 2 | Credentials | Reported on title page |
| 3 | Occupation | Reported: “Both researchers are experienced mental health nurses…” *(Method 3.5)* |
| 4 | Gender | **Reported** (Method 3.5) |
| 5 | Experience and training | Reported: “combined forty-five years… adult & CAMHS… academia”; insider positionality; one author with lived experience *(Method 3.5)* |

**Relationship with participants**

| **No.** | **Item** | **Where reported** |
| --- | --- | --- |
| 6 | Relationship established | “no prior relationship” *Method 3.3* |
| 7 | Participant knowledge of the interviewer | Reported: aims implied; consent process stated. Participants received an information sheet describing aims/research team*(Ethics 3.8; Supplementary File Q1 Consent)* |
| 8 | Interviewer characteristics (bias/assumptions/interests) | Reported via insider positionality + reflexive awareness *(Method 3.5; Rigour 3.7)* |

**Domain 2: Study design**

**Theoretical framework**

| **No.** | **Item** | **Where reported** |
| --- | --- | --- |
| 9 | Methodological orientation and theory | Interpretive description (Thorne, 2016) guiding inductive analysis *(Method 3.2; 3.5)* |

**Participant selection**

| **No.** | **Item** | **Where reported** |
| --- | --- | --- |
| 10 | Sampling | Convenience + snowball *(Method 3.3)* |
| 11 | Method of approach | Professional networks + organisational newsletters/staff bulletins; online survey distribution *(Method 3.3)* |
| 12 | Sample size | 116 practitioners *(Abstract; Results; Table 1)* |
| 13 | Non-participation | Reported as not calculable: unknown population, no response rate *(Method 3.4)* (You can also state: “dropout/refusal not tracked due to anonymous survey”) |

**Setting**

| **No.** | **Item** | **Where reported** |
| --- | --- | --- |
| 14 | Setting of data collection | UK mental health services, multiple sites in the North of England; online survey *(Method 3.3–3.4)* |
| 15 | Presence of non-participants | **N/A** – online survey *(Method 3.4)* |
| 16 | Description of sample | Table 1 demographics + professional roles *(Results; Table 1)* |

**Data collection**

| **No.** | **Item** | **Where reported** |
| --- | --- | --- |
| 17 | Interview guide | **Adapt for survey**: survey developed from scoping review; free-text questions listed in Supplementary File *(Method 3.4; Supplementary File 1)* |
| 18 | Repeat interviews | **N/A** *(Design: online survey)* |
| 19 | Audio/visual recording | **N/A** *(Design: online survey)* |
| 20 | Field notes | **N/A** |
| 21 | Duration | **Reported in Abstract** |
| 22 | Data saturation | **Not reported** as saturation. |
| 23 | Transcripts returned | **N/A** *(Design: survey free-text)* |

**Domain 3: Analysis and findings**

**Data analysis**

| **No.** | **Item** | **Where reported** |
| --- | --- | --- |
| 24 | Number of data coders | First author developed initial codes; themes refined with second author *(Method 3.5)* |
| 25 | Description of coding tree | Not reported. |
| 26 | Derivation of themes | Inductive, interpretive description; latent meanings aligned to aim *(Method 3.5)* |
| 27 | Software | **Not reported** (Excel) |
| 28 | Participant checking | **N/A** (online anonymous survey; no member checking reported) |

**Reporting**

| **No.** | **Item** | **Where reported** |
| --- | --- | --- |
| 29 | Quotations presented | Yes—multiple quotations with role identifiers (e.g., “Mental health nurse 3”) *(Results 4.1–4.3)* |
| 30 | Data and findings consistent | Themes illustrated with quotes; Tables 2 + narrative links *(Results; Table 2)* |
| 31 | Clarity of major themes | Three themes + one subtheme named and defined *(Results; Table 2)* |
| 32 | Clarity of minor themes/diverse cases | Some diversity is present (disagreement about “no stable home - no intervention”), but you don’t explicitly label “minor” or “deviant” cases. *(Results 4.3; Discussion)* |
